# Environmental Benzopyrene Attenuates Stemness of Placenta-Derived Mesenchymal Stem Cells via Aryl Hydrocarbon Receptor

**DOI:** 10.1155/2019/7414015

**Published:** 2019-01-13

**Authors:** June Seok Heo, Ja-Yun Lim, Sangshin Pyo, Dae Wui Yoon, Dongsook Lee, Wen Xiu Ren, Seung Gwan Lee, Gi Jin Kim, Jinkwan Kim

**Affiliations:** ^1^Department of Integrated Biomedical and Life Sciences, College of Health Science, Korea University, Seoul 03722, Republic of Korea; ^2^Obstructive Upper Airway Research Laboratory, Department of Pharmacology, Seoul National University College of Medicine, Seoul 03722, Republic of Korea; ^3^Department of Radiology, The Affiliated Hospital of Southwest Medical University, Luzhou, Sichuan Province 400700, China; ^4^Department of Health and Environmental Science, College of Health Science, Korea University, Seoul 03722, Republic of Korea; ^5^Department of Biomedical Science, CHA University, Seongnam 13488, Republic of Korea; ^6^Department of Biomedical Laboratory Science, College of Health Science, Jungwon University, Chung-buk 28024, Republic of Korea

## Abstract

The toxic effects of particulate matter have been linked to polycyclic aromatic hydrocarbons (PAHs) such as benzopyrene. PAHs are potent inducers of the aryl hydrocarbon receptor (AhR), which is an expressed nuclear receptor that senses environmental stimuli and modulates gene expression. Even though several studies have shown that the benzopyrene (BP) of chemical pollutants significantly impaired stem cell activity, the exact molecular mechanisms were not clearly elucidated. In the present study, we aimed to investigate the effects of BP on placenta-derived mesenchymal stem cells (PD-MSCs) *in vitro*. We found that the AhR in PD-MSCs was expressed under the treatment of BP, and its activation markedly disrupted osteogenic differentiation through the alteration of stemness activity of PD-MSCs. Moreover, BP treatment significantly reduced the proliferation activity of PD-MSCs and expression of pluripotent markers through the induction of AhR. Treatment with StemRegenin 1 (SR1), a purine derivative that antagonizes the AhR, effectively prevented BP-induced reduction of the proliferation and differentiation activity of PD-MSCs. In this study, we found that BP treatment in PD-MSCs markedly obstructs PD-MSC stemness through AhR signaling. Noteworthy, SR1-mediated MSC application will contribute to new perspectives on MSC-based therapies for air pollution-related bone diseases.

## 1. Introduction

Environmental pollutants are associated with the increased risk of various health problems such as cancer as well as cardiovascular and/or pulmonary diseases in humans. Polycyclic aromatic hydrocarbons (PAHs) of ambient pollutants are a class of chemicals produced from the incomplete combustion of organic materials [1]. Many PAHs found in our environment have toxic, carcinogenic, and mutagenic properties. PAHs are known potent agonists of the aryl hydrocarbon receptor (AhR), a ligand-activated transcription factor involved in the modulation of biological responses to aromatic hydrocarbons [2]. AhR activation leads to the inhibition of growth and differentiation of endometrial epithelial cells and liver progenitor cells, and the AhR modulates cellular function, including cell death, growth, and differentiation [3, 4].

Benzopyrene (BP) is a PAH found in tobacco smoke, coal tar, grilled meats, and many foods. BP, one of the most intensively studied pollutants, has been considered a potentially carcinogenic substance and an important risk factor for cardiovascular diseases [5, 6]. In addition, environmental pollution, such as BP from cigarette smoking, has momentous damages on the stemness and proliferation of various stem cells [7–9]. Based on previous studies, BP is thought to exert its effect via AhR activation, leading to uncontrolled changes [10]. BP has also been shown to influence the expression of genes related to cell regulation and exert inhibitory effects of adipogenesis through AhR in mouse 3T3 cells [11–13].

Toxicology is defined as the study of adverse effects that occur in living organisms due to chemical substances. Recently, toxicology testing using stem cells has been of interest because they can be theoretically cultured indefinitely in an undifferentiated state without cell immortalization [14]. In other words, stem cells are a valuable tool and an *in vitro* model system to screen new drugs and toxic chemicals because they participate in the regeneration of damaged tissues *in vivo* and can differentiate into diverse cell types depending on the specific conditions. In this regard, adult stem cells derived from various sources, such as human bone marrow, adipose tissue, and placenta, have been continuously employed to test reproductive toxicity, hepatotoxicity, cardiotoxicity, and genotoxicity [15].

Mesenchymal stem cells (MSCs) obtained from the placenta, adipose tissue, or bone marrow are multipotent adult stem cells capable of differentiating into osteocytes, chondrocytes, and adipocytes under proper conditions *in vitro* [16]. Recently, MSCs are being used for various clinical applications because they reside in various tissues to repair them when damaged [17]. Since MSCs have a longer lifespan, they are vulnerable to the long-term toxic effects of chemical or environmental agents such as BP. Yu et al. reported that the upregulation of AhR inhibited the proliferation and differentiation of osteoblasts [18]. AhR, known to be related to the phenotypic effects of PAHs, has been shown to suppress adipogenesis in human bone marrow MSCs by BP treatment [19]. However, whether AhR by BP influences the functionality of human MSCs has not been explored. Therefore, it is worthwhile to investigate the cellular effects of BP and to understand the underlying mechanisms regarding the signaling pathways affected by BP and its effects on MSC functionality.

Over the last decade, the placenta has been considered a promising source of MSCs for stem cell-based therapies because of their noninvasive acquisition methods, avoidance of ethical issues, and accessibility [20]. In general, the placenta is known to have an excellent transporter system of substances as well as selective transmission of toxic molecules for the safe growth of the baby during pregnancy. Therefore, it is known that PD-MSCs have such placental characteristics and are well expressed in various transporter systems. In previous reports, Lee et al. demonstrated that the expression patterns of ABCG transporter systems might be involved in the sensitivity of PD-MSCs to several liver toxic chemicals regardless of hepatogenic differentiation compared to bone marrow-derived MSCs [21, 22].

StemRegenin 1 (SR1), a cell-permeable purine derivate, has been shown to promote self-renewal of hematopoietic stem cells by inhibiting the AhR [23]. However, the role of SR1 in MSCs is unknown. This led us to evaluate the impact of SR1 on AhR-expressed MSCs. In the present study, we aimed to investigate the effects of BP on PD-MSCs *in vitro*. We isolated and cultured PD-MSCs from the placenta and investigated the expression of AhR signaling and pluripotent/multipotent markers to further explore the potential protective methods for preserving PD-MSC stemness from long-lasting air pollutants including BP by inhibiting the expression of AhR using SR1.

## 2. Materials and Methods

### 2.1. Culture of Placenta-Derived MSCs

Full-term normal human placentas were obtained from healthy donors after informed consent. This study was approved by the Institutional Review Boards of CHA General Hospital, Seoul, Korea (07-18). As previously described, mononuclear cells were isolated from the chorionic plate of the placenta, and the cells were cultivated by the plastic adherence method [24]. The cells were cultured in DMEM with low glucose supplemented with 10% fetal bovine serum (FBS) and 1% penicillin/streptomycin (P/S) at 37°C with 5% CO_2_ (Invitrogen, Carlsbad, CA, USA). The medium was changed with 1 *μ*g/mL heparin (Sigma-Aldrich, St. Louis, MO, USA) and 25 *μ*g/mL FGF4 (PeproTech Inc., NJ) every 3 or 4 days. The PD-MSCs were replated using 0.05% trypsin/EDTA (Invitrogen) when they reached approximately 80–90% confluence. Mesenchymal stem cells from 3 different donors were used under passage 10. BP was dissolved in DMSO, and PD-MSCs were cultured for 24 h for short-term experiment and for 7 days for long-term experiment. In all experiments, when BP was treated, it was labeled as BP(+) and untreated control was marked as BP(−). Also, if StemRegenin 1 (SR1, STEMCELL Technologies, Vancouver, Canada) was added to the cells, it was labeled as SR1(+). 1 *μ*M of SR1 was treated whenever the medium was changed.

### 2.2. RT-PCR

Total RNA was extracted by a RiboEx reagent (GeneAll, Seoul, Korea). Standard reverse transcription was performed using a Maxime™ RT PreMix (iNtRON, Seongnam, Korea). RT-PCR assay was performed by using PCR primers (Bioneer, Daejeon, Korea) with the conditions listed in Table 1. The amplification program consisted of 24–35 cycles of the following parameters: 94°C for 30 s; annealing at 62°C for 30 s, and extension at 72°C for 30 s, followed by a final amplification step for 10 min 72°C. The levels of the different PCR targets generated by PCR cycles were confirmed using Bio-Imaging Systems (DNR Bio-Imaging Systems Ltd., Neve Yamin, Israel). The level of glyceraldehyde 3-phosphate dehydrogenase (*GAPDH*) was used as an internal control. One representative of 3 independent experiments is shown. The data are expressed as the mean ± SD of three independent experiments.

### 2.3. Enzyme-Linked Immunosorbent Assay (ELISA)

The human AhR concentrations were determined by ELISA. Briefly, cells were harvested after BP treatment for 7 days and transferred to a 1.5 mL tube. Then, the cells were stored overnight at −20°C. After two freeze-thaw cycles, the cell lysates were centrifuged for 5 min at 5000 ×*g* at 4°C. Cell lysates were assayed immediately by a human AhR ELISA kit (MyBioSource, San Diego, USA) according to the manufacturer's protocols. The data are expressed as the mean ± SD of three independent experiments.

### 2.4. Cell Proliferation Assay

Cells at passage 8 were seeded at a density of 1 × 10^3^/well in 96-well plates (BD Falcon, USA) for the analysis of cell proliferation. When the medium was changed, BP was added to each well of the culture plate. A proliferation assay was performed by using the EZ-CYTOX kit (Daeil Lab, Seoul, Korea). EZ-CYTOX contains WST, which when dissolved in water produces a water-soluble formazan by succinate-tetrazolium reductase. EZ-CYTOX, being nonradioactive, allows colorimetric assays for the determination of the number of viable cells during cell growth. Cells were cultured for 7 days and then analyzed according to the manufacturer's instructions. The data are expressed as the mean ± SD of three independent experiments.

### 2.5. *β*-Galactosidase Assay

PD-MSCs at passage 9 were cultured for 4 weeks, and *β*-galactosidase assay was performed when the cells displayed senescence phenotype. A senescence detection kit (BioVision Inc., CA, USA) was used to detect *β*-galactosidase activity in senescent cells according to the manufacturer's protocols. Briefly, cultured cells were washed with phosphate-buffered saline (PBS) (Invitrogen) and fixed at room temperature. After washing with PBS twice, cells were incubated with *β*-galactosidase staining solution overnight at 37°C. The number of *β*-galactosidase-stained cells was counted under a microscope (Olympus-IX71, Olympus, Tokyo, Japan). One representative of 3 independent experiments is shown. The data are expressed as the mean ± SD of three independent experiments.

### 2.6. Immunostaining

After washing with PBS containing 0.1% BSA (Sigma-Aldrich), cells were fixed in 4% paraformaldehyde solution (Biosesang, Seongnam, Korea) at 4°C. After 20 min, cells were washed with PBS containing 0.1% BSA followed by permeabilization solution containing PBS with 0.1% BSA and 0.3% Triton X-100 (Sigma-Aldrich). After 10 min, antibodies were added at the following dilutions: human AhR (Abcam, Cambridge, MA, USA) 1 : 200 and Annexin V (Millipore, Burlington, USA) 1 : 200. Next, PBS containing 0.1% BSA and 10% FBS was dispensed to the cells. The cells were incubated overnight at 4°C. Then, fluorescence-labeled secondary antibodies were used as follows: anti-rabbit IgG 594 (1 : 400) (Invitrogen) and anti-mouse IgG 488 (1 : 400) (Invitrogen). After washing, cells were costained with 4′6-diamidino-2-phenylindole (DAPI) at 1 : 1000 (Sigma-Aldrich) for 6 min. Fluorescence images were taken using a Confocal Laser Scanning Microscope (Carl-Zeiss LSM 700 Exciter, Oberke, Germany). One representative of 3 independent experiments is shown.

### 2.7. Colony-Forming Unit (CFU-F) Assay

To assess the self-renewal capacity of PD-MSCs, a CFU-F assay was performed. Briefly, 1 × 10^3^ cells at passage 8 were seeded in 100 mm plates (Corning Inc., Corning, NY, USA), and the cells were maintained for 14 days. Whenever the medium was changed every 3 days, BP was added to the test group. Following culture for 14 days, the cells were washed with PBS. Next, the cells were stained with 3% crystal violet (Sigma-Aldrich) for 10 min at room temperature, and then the stained colonies were counted. The data are expressed as the mean ± SD of three independent experiments.

### 2.8. Differentiation Assay

To differentiate PD-MSCs into osteocytes (PT-3002), chondrocytes (PT-3003), and adipocytes (PT-3004), the cells at passage 8 were maintained in osteogenic differentiation medium, chondrogenic differentiation medium, and adipogenic differentiation medium for 3 weeks (Cambrex, Lonza, MD, USA), respectively. The medium was replaced every 3 days, and 10 ng/mL of transforming growth factor- (TGF-) *β*3 (Cambrex, PT-4124) was added to the cells in order to differentiate the chondrocytes whenever the medium was changed. The differentiated cells were stained with silver nitrate solution to determine osteogenesis, safranin O solution to assess chondrogenesis, and oil red O solution to evaluate adipogenesis. Images of the stained cells were taken using an inverted phase microscope (Olympus-IX-71). One representative of 3 independent experiments is shown.

### 2.9. Growth Factor Assay

PD-MSCs at passage 8 were cultured in culture medium and 1 *μ*M BP with culture medium for 7 days, respectively. The cultured conditioned medium was used as a sample for growth factor assay. The supernatant from the control and test group was harvested, and the supernatant was combined with coated beads from a human premixed multianalyte kits (R&D Systems, Minneapolis, USA) for hepatocyte growth factor (HGF), platelet-derived growth factor (PDGF), and vascular endothelial growth factor (VEGF). Samples were assayed in duplicate and analyzed using a Luminex according to the instructions of the manufacturer. The concentrations were determined from an appropriate standard curve. Concentrations of TGF-*β*1 were quantified by a human TGF-*β*1 ELISA kit (KOMA BIOTECH Inc., Seoul, Korea) according to the manufacturer's protocols. The data are expressed as the mean ± SD of three independent experiments.

### 2.10. Statistical Analysis

Data are expressed as the means ± standard deviation (SD). Statistical comparisons were performed by a *t*-test and one-way analysis of variance (ANOVA), followed by post hoc Bonferroni corrections. The differences were considered statistically significant at *P* < 0.05. Statistical analyses were performed using SPSS software (SPSS Inc., Chicago, IL, USA).

## 3. Results

### 3.1. Expression of AhR in PD-MSCs

**Figure 1:**
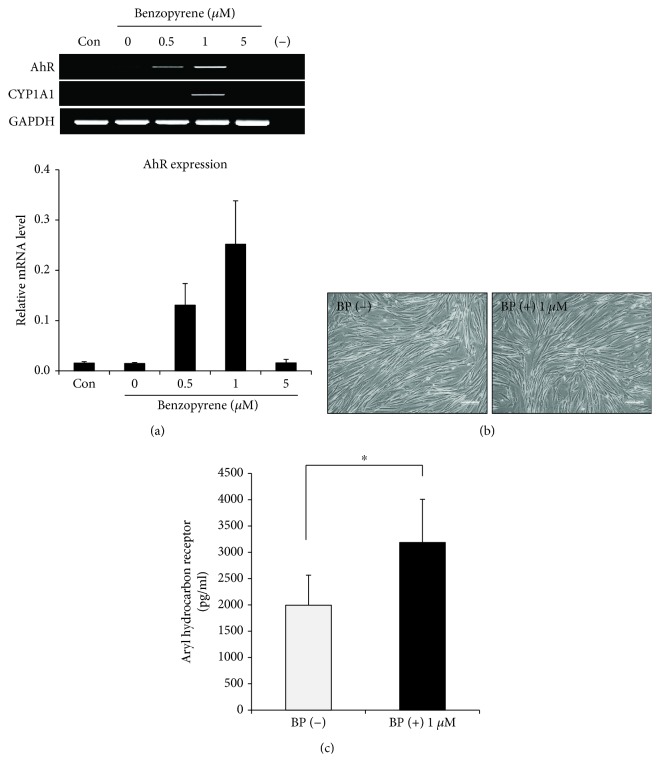
Expression of the aryl hydrocarbon receptor (AhR) in placenta-derived mesenchymal stem cells (PD-MSCs). (a) PD-MSCs were cultured with BP for 7 days, and mRNA was extracted. Expression of AhR was evaluated by RT-PCR. One representative of 3 independent experiments is shown. Expression levels relative to GAPDH are shown. (−) indicates a negative control using water alone. (b) Phase contrast images were obtained before and after treatment of 1 *μ*M BP for 7 days (scale bar, 200 *μ*m; magnification, 100x). One representative of 3 independent experiments is shown. (c) Cell lysates were collected after PD-MSCs were cultured with 1 *μ*M BP for 7 days. AhR concentration in cultured PD-MSCs was confirmed by ELISA. The data are expressed as the mean ± SD of three independent experiments. ^∗^*P* < 0.05.

BP, a polycyclic aromatic hydrocarbon, binds to the AhR in the cytosol. The AhR that is present in the cytoplasm is activated upon ligand binding to it. The activated AhR has been shown to be one of the modulators in diverse cell types. Therefore, we evaluated the expression of AhR after BP treatment (0.5, 1, and 5 *μ*M) in PD-MSCs. The PD-MSCs displayed the highest expression level of AhR with 1 *μ*M BP, as shown in Figure 1(a). Notably, the expression of AhR was inhibited with 5 *μ*M BP. These results revealed that BP does not have a dosage-dependent effect on PD-MSCs. CYP1A1 has been known as a target gene of the AhR pathway. We examined mRNA expression of CYP1A1 in PD-MSCs treated with BP for 7 days. We confirmed that the expression of CYP1A1 mRNA was markedly induced in 1 *μ*M BP-treated PD-MSCs (Figure 1(a)). In order to determine whether the results are specific to PD-MSCs, bone marrow-derived mesenchymal stem cells (BM-MSCs) were tested under the same conditions. Similarly, the AhR gene in the BM-MSCs was induced by treatment with 1 *μ*M BP (data not shown). In accordance with these results, 1 *μ*M BP was used to further study the effects of BP on PD-MSCs. By morphological observations, there were no significant differences despite the BP treatment (Figure 1(b)). To confirm the expression of AhR by BP, we quantified the AhR expression using whole cell lysates by ELISA. The concentrations of AhR were significantly increased in 1 *μ*M BP-treated PD-MSCs over not treated (*p* = 0.048, Figure 1(c)). These data show that BP plays a role in inducing AhR signaling.

### 3.2. Effect of BP on the Proliferative Activity of PD-MSCs

**Figure 2:**
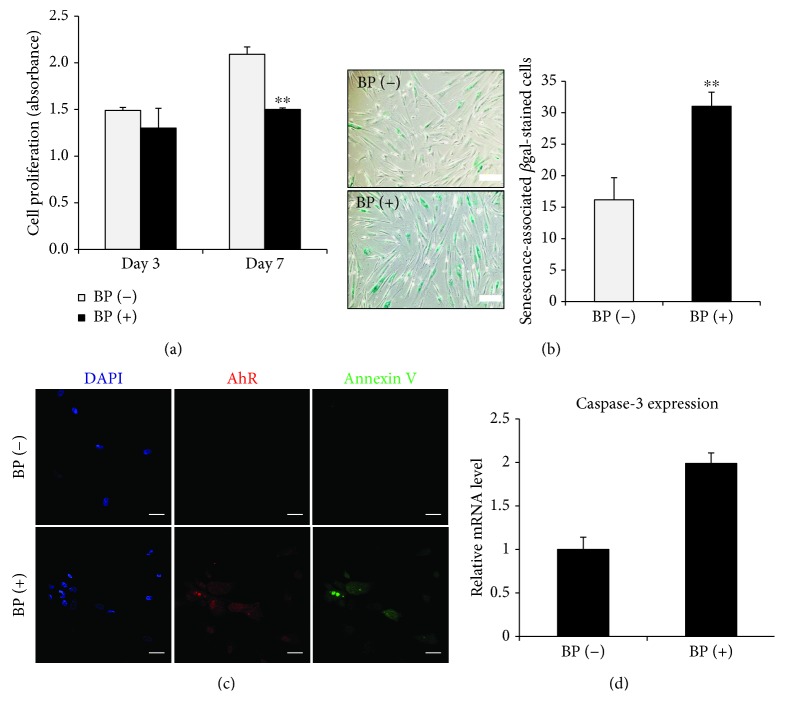
The effect of BP on placenta-derived mesenchymal stem cell (PD-MSC) activity. (a) PD-MSCs were cultured with 1 *μ*M BP for 7 days. Growth activity was measured using WST-based EZ-CYTOX at days 3 and 7, respectively. The data are expressed as the mean ± SD of three independent experiments. ^∗∗^*P* < 0.01. (b) Senescence was confirmed by *β*-galactosidase staining after long-term culture (4 weeks). One representative of three independent experiments is shown (scale bar, 200 *μ*m; magnification, 100x). The number of *β*-galactosidase-stained cells was enumerated. The data are expressed as the mean ± SD of three independent experiments. ^∗∗^*P* < 0.01. (c) Immunostaining was performed to evaluate apoptosis and aryl hydrocarbon receptor (AhR) expression in PD-MSCs cultured with 1 *μ*M BP for 7 days (scale bar, 20 *μ*m; magnification, 200x). One representative of 3 independent experiments is shown. (d) RT-PCR analysis of 1 *μ*M BP-treated PD-MSCs for 7 days revealed enhanced mRNA expression of caspase-3. The data are expressed as the mean ± SD of three independent experiments.

To investigate the effects of BP on cultures of PD-MSCs, the growth rate was evaluated 3 and 7 days after exposure to 1 *μ*M BP, respectively. No significant changes were observed by the BP treatment for 3 days, while BP reduced the proliferation rate of PD-MSCs after 7 days as compared to the control group (*p* < 0.01, Figure 2(a)). The results suggested that BP inhibited the growth activity of the PD-MSCs by accumulating the effect of BP within cells. We next evaluated whether BP induces PD-MSC senescence after long-term exposures to BP. The cells were replated whenever they reached 90% confluence. PD-MSCs were stained with *β*-galactosidase when they exhibited morphological patterns of typical senescence such as an aggregated phenotype. Interestingly, BP-treated PD-MSCs had a significant increase in the percentage of *β*-galactosidase-stained cells compared to PD-MSCs in the control group (*p* = 0.003, Figure 2(b)). These results confirm that BP has toxic effects associated with the replicative senescence of PD-MSCs.

In order to determine whether senescent cells treated by BP undergo apoptosis, immunostaining assay was utilized to identify the expression of AhR and Annexin V. A few cells were coexpressed for AhR and Annexin V in the BP-treated group after 7 days of culture (Figure 2(c)). It was well known that caspase-3 is involved in the induction of apoptosis. To confirm apoptosis in PD-MSCs after treatment with BP, mRNA levels of caspase-3 were measured by a PCR assay. The results showed that upregulation of caspase-3 was observed in the BP-treated group (Figure 2(d)). Together, these results indicated that BP promoted PD-MSC senescence with growth arrest and partial activation of apoptosis.

### 3.3. Effect of BP on PD-MSC Stemness

**Figure 3:**
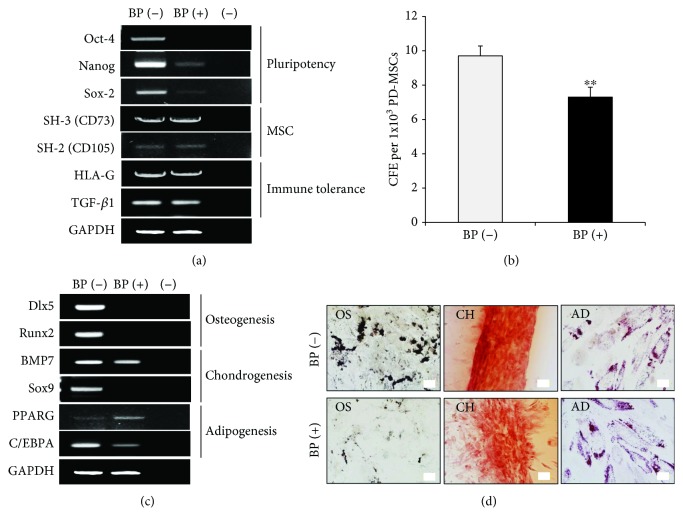
The effect of BP on stemness in placenta-derived mesenchymal stem cells (PD-MSCs). (a) Gene expression of 1 *μ*M BP-treated PD-MSCs for 7 days was analyzed by RT-PCR for pluripotency, MSC, and immune tolerance markers compared to control. One representative of 3 independent experiments is shown. (b) Self-renewal capacity of 1 *μ*M BP-treated PD-MSCs (passage 8) for 7 days was evaluated by a CFU-F assay. The number of colonies was counted after 14 days. The data are expressed as the mean ± SD of three independent experiments. ^∗∗^*P* < 0.01. (c) RT-PCR analysis of multipotency-related genes was performed in the control and 1 *μ*M BP-treated PD-MSCs for 7 days. One representative of 3 independent experiments is shown. (d) Differentiation for osteogenesis, adipogenesis, and chondrogenesis was induced for 2 weeks. 1 *μ*M BP was added to the induction medium whenever the medium was changed. Osteogenic differentiation was examined by von Kossa staining (scale bar, 100 *μ*m; magnification, 200x). Adipogenic differentiation was determined by oil red O staining (scale bar, 50 *μ*m; magnification, 400x). Chondrogenesis was evaluated by safranin O staining (scale bar, 100 *μ*m; magnification, 200x). One representative of 3 independent experiments is shown. In RT-PCR, (−) indicates a negative control using water alone.

To assess the self-renewal activity of PD-MSCs, we analyzed the expression levels of pluripotency markers in the BP-treated PD-MSCs compared to control PD-MSCs. Oct-4, Nanog, and Sox2, key transcription factors essential in maintaining the pluripotency of embryonic stem cells, were significantly repressed in BP-treated PD-MSCs (Figure 3(a)). SH-3(CD73) and SH-2(CD105) genes specific for MSCs were similarly expressed in all groups (Figure 3(a)). Furthermore, no significant differences were detected in the expression of HLA-G and TGF-*β*1 genes related to immunomodulation (Figure 3(a)). We subsequently performed a CFU-F assay in order to identify the effects of BP on the downregulation of pluripotency markers. BP-treated PD-MSCs had decreased self-renewal capacity compared with that of the control group (*p* = 0.008, Figure 3(b)). We next investigated the changes in MSC multipotency including osteogenesis, chondrogenesis, and adipogenesis at the mRNA level. Distal-less homeobox 5 (Dlx5) and runt-related transcription factor 2 (Runx2) known as regulators for osteogenic differentiation were markedly suppressed by BP treatment at the mRNA level (Figure 3(c)). Adipogenesis-related peroxisome proliferator-activated receptor gamma (PPARG) and CCAAT/enhancer binding protein alpha (C/EBPA) genes were expressed similarly in all groups, while the expression level of the transcription factor Sox9 for chondrogenesis was inhibited in BP-treated PD-MSCs (Figure 3(c)). These results indicated that alterations of transcription factors for mesenchymal lineages may trigger substantial changes in the *in vitro* differentiation assay.

To further assess the effects of BP in PD-MSC differentiation, osteoblastic, chondrogenic, and adipogenic differentiation assays were performed. BP-treated PD-MSCs showed lower amounts of von Kossa staining, which detects calcium for osteogenic differentiation, compared to that of controls, whereas there were no differences in safranin O staining for chondrogenesis and oil red O staining for adipogenesis in both groups (Figure 3(d)). These results imply that BP has specific negative effects on the osteogenesis of PD-MSCs by downregulating the expression of Dlx5 and Runx2 genes.

**Figure 4:**
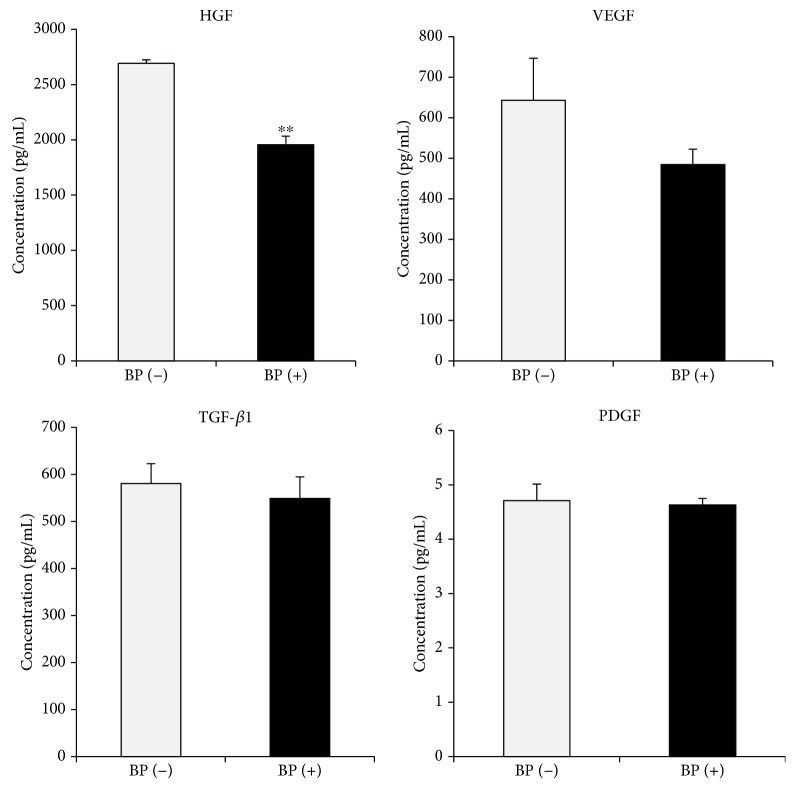
Growth factor assay shows a reduction in cytokine concentration in 1 *μ*M BP-treated placenta-derived mesenchymal stem cells (PD-MSCs) for 7 days. The data are expressed as the mean ± SD of three independent experiments. ^∗∗^*P* < 0.01.

MSCs secrete paracrine factors that exert wound-healing effects as well as a protective effect on the heart [25, 26]. Recent studies have shown that paracrine factors including exosomes, microvesicles, and secretomes from MSCs could provide a promising strategy for the treatment of various diseases [27]. In this regard, we examined paracrine factors such as HGF, VEGF, TGF-*β*1, and PDGF released from PD-MSCs. TGF-*β*1 and PDGF for growth and differentiation of MSCs were similarly measured, whereas VEGF, an important angiogenesis factor, was decreased by BP treatment, although no significant differences were observed (Figure 4). Notably, HGF-promoting proliferation was significantly reduced in BP-treated PD-MSCs (*p* = 0.001, Figure 4). These results indicated that BP inhibits beneficial paracrine factors released from PD-MSCs by suppressing activity as shown in previous data.

### 3.4. A Protective Effect of SR1 on BP-Treated PD-MSCs

**Figure 5:**
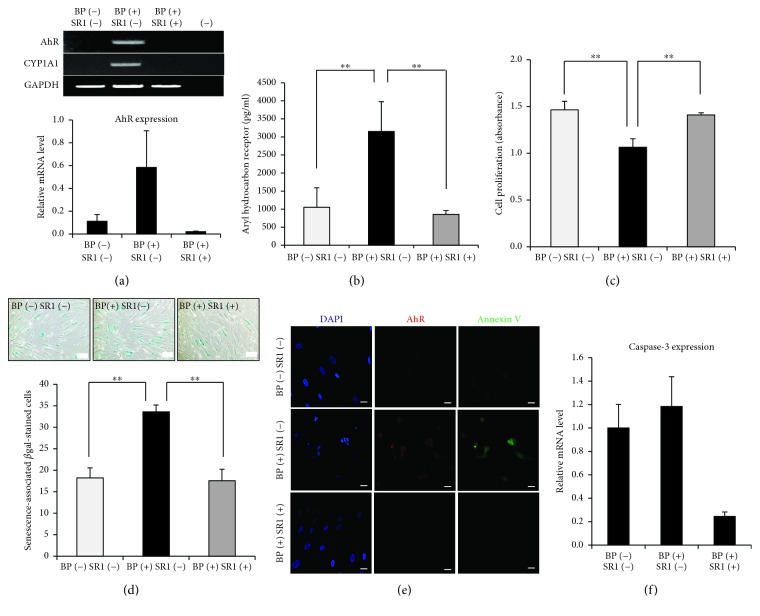
StemRegenin 1 (SR1) maintains placenta-derived mesenchymal stem cell (PD-MSC) activity and decreases BP-induced apoptosis. (a) The expression levels of AhR were determined by RT-PCR after treatment of 1 *μ*M BP and 1 *μ*M BP with 1 *μ*M SR1 for 7 days, respectively. One representative of 3 independent experiments is shown. Expression levels relative to GAPDH are shown. (−) indicates a negative control using water alone. (b) The concentration of AhR was analyzed by ELISA after treatment of 1 *μ*M BP and 1 *μ*M BP with 1 *μ*M SR1 for 7 days, respectively. The data are expressed as the mean ± SD of three independent experiments. ^∗∗^*P* < 0.01. (c) The proliferative activity of PD-MSCs was measured by EZ-CYTOX after treatment of 1 *μ*M BP and 1 *μ*M BP with 1 *μ*M SR1 for 7 days, respectively. The data are expressed as the mean ± SD of three independent experiments. ^∗∗^*P* < 0.01. (d) Culture of PD-MSCs with 1 *μ*M SR1 prevents cellular senescence (scale bar, 200 *μ*m; magnification, 100x). One representative of 3 independent experiments is shown. The number of *β*-gal positive cells was enumerated after 4 weeks. The data are expressed as the mean ± SD of three independent experiments. ^∗∗^*P* < 0.01. (e) Confocal microscopy images were collected after treatment of 1 *μ*M BP and 1 *μ*M BP with 1 *μ*M SR1 for 7 days, respectively. One representative of 3 independent experiments is shown (scale bar, 20 *μ*m; magnification, 200x). (f) RT-PCR analysis revealed diminished expression of caspase-3 in treatment of 1 *μ*M BP with 1 *μ*M SR1. The data are expressed as the mean ± SD of three independent experiments.

SR1, a potent antagonist of AhR signaling, promotes the expansion and differentiation of hematopoietic stem cells [23]. We next applied SR1 to inhibit AhR expression by BP. After 7 days, morphological changes were not observed in any conditions including BP-treated SR1 (hereafter designated as BP[+] SR1[+]). A PCR assay revealed that BP-treated PD-MSCs expressed the AhR gene, while the expression of AhR was strongly suppressed in BP-treated SR1 PD-MSCs (Figure 5(a)). Moreover, we detected inhibition of CYP1A1 mRNA in SR1-treated PD-MSCs after exposure to BP for 7 days (Figure 5(a)). A decrease in AhR expression by SR1 was subsequently confirmed using ELISA (BP[−] SR1[−], BP[+] SR1[−], *p* = 0.002; BP[+], BP[+] SR1[+], *p* = 0.001; Figure 5(b)). These results indicated that SR1 acts as a powerful inhibitor of AhR in PD-MSCs. Furthermore, cultivation of PD-MSCs using SR1 increased the number of PD-MSCs compared to that of BP-treated PD-MSCs by inhibiting AhR expression (BP[−] SR1[−], BP[+] SR1[−], *p* = 0.002; BP[+] SR1[−], BP[+] SR1[+], *p* = 0.004; Figure 5(c)).

We next examined whether SR1 inhibited senescence of BP-treated PD-MSCs. PD-MSCs cultured with SR1 displayed a significant decrease in the percentage of *β*-galactosidase-positive PD-MSCs compared to BP-treated PD-MSCs. To quantitatively evaluate replicative senescence, the number of *β*-galactosidase-stained cells was counted after 14 days (BP[−] SR1[−], BP[+] SR1[−], *p* < 0.001; BP[+] SR1[−], BP[+] SR1[+], *p* < 0.001; Figure 5(d)). As shown in Figure 2(c), BP induced apoptosis of some PD-MSCs by upregulation of AhR, as confirmed by the expression of Annexin V and caspase-3 in BP-treated PD-MSCs. Therefore, we tested whether AhR inhibition by SR1 prevents apoptosis in BP-treated PD-MSCs. Surprisingly, SR1 suppressed the expression of AhR and Annexin V by BP treatment (Figure 5(e)), and the inhibition of the caspase-3 gene was confirmed by PCR analysis (Figure 5(f)). These results show that SR1-treated PD-MSCs maintained normal growth kinetics by inhibiting cellular senescence and apoptosis.

**Figure 6:**
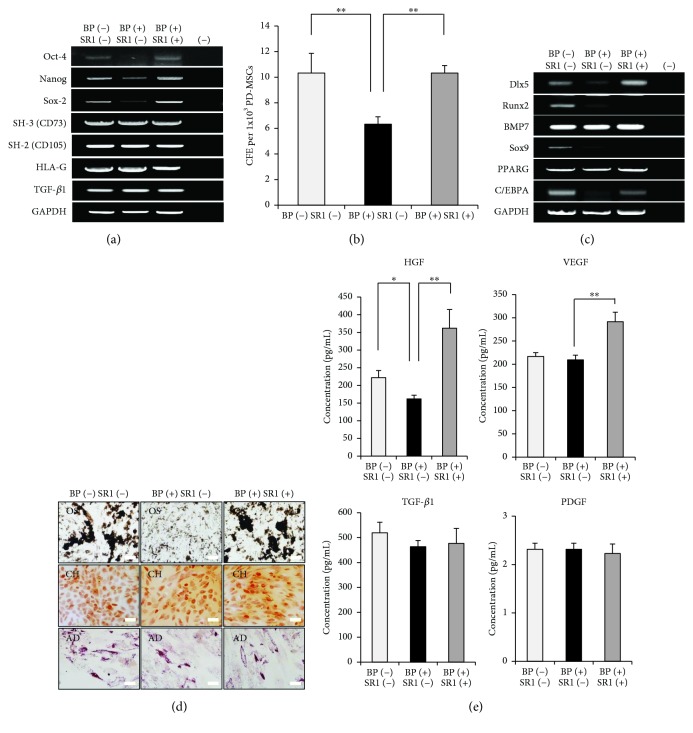
Culture with StemRegenin 1 (SR1) restores stemness of damaged placenta-derived mesenchymal stem cells (PD-MSCs). (a) RT-PCR analysis shows that 1 *μ*M SR1 improves the expression of Oct-4, Nanog, and Sox-2 in 1 *μ*M BP-treated PD-MSCs. One representative of 3 independent experiments is shown. (b) Stemness was evaluated by a CFU-F assay. PD-MSCs treated with 1 *μ*M BP and 1 *μ*M BP with 1 *μ*M SR1 were cultured for 14 days at low density. The data are expressed as the mean ± SD of three independent experiments. ^∗∗^*P* < 0.01. (c) RT-PCR analysis for osteogenesis, chondrogenesis, and adipogenesis was performed in the control, 1 *μ*M BP-treated, and 1 *μ*M BP with 1 *μ*M SR1-treated groups. One representative of 3 independent experiments is shown. (d) The differentiation capacity of PD-MSCs was examined for osteogenesis and chondrogenesis (scale bar, 100 *μ*m; magnification, 200x) and for adipogenesis (scale bar, 50 *μ*m; magnification, 400x) after treatment of 1 *μ*M BP and 1 *μ*M BP with 1 *μ*M SR1 for 14 days. One representative of 3 independent experiments is shown. (e) The concentration of growth factors released from PD-MSCs was measured after treatment of 1 *μ*M BP and 1 *μ*M BP with 1 *μ*M SR1 for 7 days. The data are expressed as the mean ± SD of three independent experiments. In RT-PCR, (−) indicates a negative control using water alone. ^∗^*P* < 0.05 and ^∗∗^*P* < 0.01.

Next, we asked whether SR1 enhances the self-renewal capacity of BP-treated PD-MSCs. Interestingly, reduced expression of Oct-4, Nanog, and Sox2 were upregulated in BP-treated PD-MSCs by SR1 (Figure 6(a)). Furthermore, the BP[+] SR1[+] group displayed similar self-renewal capacity with the control group despite long-term culture with BP (BP[−] SR1[−], BP[+] SR1[−] *p* = 0.008; BP[+] SR1[−], BP[+] SR1[+], *p* = 0.008; Figure 6(b)). Subsequently, the effect of SR1 on the multilineage potentials of PD-MSCs was evaluated by analyzing the mRNA levels of genes associated with osteogenesis, chondrogenesis, and adipogenesis. SR1 significantly induced the mRNA expression of Dlx5 for osteogenic differentiation and C/EBPA for adipogenic differentiation, whereas Runx2 for osteogenesis and Sox9 for chondrogenesis were not detected in the BP-treated and BP[+] SR1[+] groups (Figure 6(c)). Using an *in vitro* differentiation assay, PD-MSCs of all groups were differentiated into chondrocytes and adipocytes regardless of the expression of Sox9 and C/EBPA (Figure 6(d)). Surprisingly, SR1 improved the osteogenic differentiation capacity of BP-treated PD-MSCs by inducing the mRNA, as revealed by the *in vitro* assay.

We then analyzed the concentrations of cytokines secreted from PD-MSCs, which included HGF, VEGF, TGF-*β*1, and PDGF. Compared to the control group, no significant differences were detected in the concentrations of TGF-*β*1 and PDGF (Figure 6(e)). Notably, an increase of HGF and VEGF in the BP[+] SR1[+] group was detected, implying that SR1 maintains and promotes the functionality of PD-MSCs (HGF; BP[−] SR1[−], BP[+] SR1[−], *p* = 0.042; BP[+] SR1[−], BP[+] SR1[+], *p* = <0.001; VEGF; BP[+] SR1[−], BP[+] SR1[+], *p* < 0.001; Figure 6(e)).

## 4. Discussion

Air pollution exerts a range of harmful effects on the human body, including the reproductive and cardiovascular systems [28]. A number of toxic effects resulting from environmental pollutants have been linked to PAHs, which contains more than one fused benzene ring. Several studies have demonstrated that PAHs, such as BP, are important determinants of genotoxicity [29]. BP, which has toxic effects, modulates transcription via the activation of the AhR [30]. The AhR expressed ubiquitously in vertebrate cells is a key factor in controlling growth activity of liver epithelial cells exposed to PAHs and mediating various cellular functions as previously reported [31]. However, the AhR-mediated effect of BP is unknown.

In this study, we supposed that the induction of AhR in PD-MSCs may be triggered by BP. We evaluated the effects of AhR agonist BP on cellular functions of PD-MSCs, with respect to cell proliferation rates, cell death rates, self-renewal capacity, secretion of growth factors, and differentiation potentials. Our results showed that 1 *μ*M of BP was suitable to upregulate the AhR pathway in PD-MSCs, showing that AhR is expressed in PD-MSCs. However, a concentration-dependent induction in AhR expression was not observed after exposure of PD-MSCs to BP. These results were similar in BM-MSCs. Whether MSCs derived various human tissues which exhibit the same results, further analysis is required. Matsunawa et al. also showed that 1 *μ*M BP conferred toxic effects via AhR-dependent induction of CYP1A1 [13, 32]. In our study, the AhR activation induced by BP has influence on the growth of PD-MSCs. A temporary 3-day exposure to BP was insufficient to act on the proliferation of PD-MSCs, while cell growth was markedly decreased after 7 days of BP treatment, indicating that continuous exposure to BP interfered with the activity of PD-MSCs. Replicative senescence is a cellular response to stress and damage [33]. PAHs including BP are toxic to tissues and organs. BP increased replicative senescence of PD-MSCs compared to PD-MSCs cultured in the absence of BP. The decrease in cell proliferation was associated with cell death due to necrosis and/or apoptosis. BP is also well known for inducing DNA damage and providing apoptotic effects in various cells [34]. The expression of Annexin V and caspase-3 did support apoptotic patterns of cell death by BP. Although BP-treated PD-MSCs have been shown to cause apoptosis, it is necessary to examine additional pathways related to cell death in further studies.

During differentiation, the induction medium stimulates the upregulation of numerous transcription factors, such as Dlx5 and Runx2 in osteogenesis, BMP7 and Sox9 in chondrogenesis, and PPARG and C/EBPA in adipogenesis. Previous studies have shown that AhR is involved in osteoblast differentiation, maturation, and mineralization, and the induction of AhR inhibited the functions of osteoblasts [18, 35]. Our data are in agreement with previous studies indicating that the AhR suppresses osteoblast proliferation and differentiation. Furthermore, the results suggested that expression of AhR might contribute to bone erosion [18]. Another study showed that BP activated AhR resulting in the inhibition of adipogenesis through PPAR*γ* [8]. Different results in AhR expression point to substantial diversity depending on cell types from various sources.

It was previously demonstrated that AhR antagonist SR1 promotes the self-renewal of human hematopoietic stem cells. One important question was whether the inhibition of AhR by SR1 could restore the functionality of PD-MSCs, such as proliferative activity, self-renewal capacity, and osteogenic differentiation potentials. Our data here revealed that SR1, an antagonist for AhR, could completely block AhR expression in PD-MSCs, effectively increasing proliferative activity, decreased cellular senescence with upregulation of pluripotency genes, and markedly reduced the number of apoptotic cells. Furthermore, SR1 reversed the inhibitory effects of BP on the osteogenic differentiation of PD-MSCs to normal levels in the PD-MSCs with BP exposure. These results are supported by a previous report that demonstrated that 3-methoxy-4-nitroflavone, another antagonist for AhR, reversed the inhibitory effects of osteogenesis [36]. Although only Dlx5, a key marker for osteogenesis, was upregulated in BP-treated PD-MSCs by SR1, the osteogenic capacity was improved similarly compared with a control group, consistent with previous data indicating that Dlx5 is a master factor during osteogenesis [37]. Our results also exhibited increased expression of growth factors, namely, HGF and VEGF, following treatment of BP-treated PD-MSCs with SR1. Taken together, SR1 restored the activity of PD-MSCs, attenuated apoptosis-induced cell damage, and enhanced stemness *in vitro*.

To our knowledge, this is the first study to demonstrate that BP exposure decreases the stemness of PD-MSCs through activation of AhR. In addition, treating PD-MSCs with SR1 significantly enhanced PD-MSCs stemness, reversed the suppression of the expression of osteogenic markers, and restored the proliferative activity and functionality in the PD-MSCs with BP exposure. Several questions regarding the BP-mediated reduced functionality in PD-MSCs remain unaddressed. Reduced activity of PD-MSCs may be due to cellular damages such as apoptosis by BP. We herein report that BP specifically inhibits osteogenesis of multilineage potentials in PD-MSCs. Furthermore, we highlight that SR1 protects PD-MSCs from BP-mediated cell damages by repressing the AhR. It is possible that other pathways might also be affected by BP exposure. Future studies are needed to uncover the mechanism of other pathways in the effect of BP exposure on PD-MSCs.

In conclusion, we have shown that AhR expression plays a critical role in regulating stemness of PD-MSCs. Applications of MSCs using small molecule compounds to activate or inactivate AhR expression will contribute greatly to stem cell-based therapies.

## Figures and Tables

**Table 1:** Primer sequences.

Gene	Primer sequence (5′-3′)	Annealing temperature (°C)	Cycle	Product size (bp)
*GAPDH*	Forward: GTGGTCTCCTCTGACTTCAACA	62	24	210
	Reverse: CTCTTCCTCTTGTGCTCTTGCT			
*AhR*	Forward: AGTCTGTTATAACCCAGACCAG	58	35	307
	Reverse: GCATCACAACCAATAGGTGTGA			
*Oct-4*	Forward: ACACTCGGACCACGTCTTTC	54	32	300
	Reverse: CGTTCTCTTTGGAAAGGTGTTC			
*Nanog*	Forward: ATAGCAATGGTGTGACGCAG	62	32	219
	Reverse: GATTGTTCCAGGATTGGGTG			
*Sox-2*	Forward: AACCAAGACGCTCATGAAGAAG	62	32	341
	Reverse: GCGAGTAGGACATGCTGTAGGT			
SH-3(CD73)	Forward: TATTGCACTGGGACATTCGGGT	62	35	443
	Reverse: GGTTGCCCATGTTGCATTCTCT			
*SH-2(CD105)*	Forward: CATCCTTGAAGTCCATGTCCTCTT	62	32	95
	Reverse: GCCAGGTGCCATTTTGCTT			
HLA-G	Forward: GCGGCTACTACAACCAGAGC	57	35	338
	Reverse: GCACATGGCACGTGTATCTC			
*TGF-β1*	Forward: GAGGTGACCTGGCCACCATT	55	32	194
	Reverse: TCCGCAAGGACCTCGGCTGG			
*Dlx5*	Forward: ACCATCCGTCTCAGGAATCG	60	32	384
	Reverse: ACCTTCTCTGTAATGCGGCC			
*Runx2*	Forward: TTGCAGCCATAAGAGGGTAG	58	32	470
	Reverse: GTCACTTTCTTGGAGCAGGA			
*BMP7*	Forward: CCAACGTCATCCTGAAGAAATAC	60	32	271
	Reverse: GCTTGTAGGATCTTGTTCATTGG			
*Sox9*	Forward: GGTTGTTGGAGCTTTCCTCA	61	32	400
	Reverse: TAGCCTCCCTCACTCCAAGA			
*PPARG*	Forward: TCTCTCCGTAATGGAAGACC	55	32	474
	Reverse: GCATTATGAGACATCCCCAC			
*CYP1A1*	Forward: TCACAGACAGCCTGATTGAG	58	35	434
	Reverse: GATGGGTTGACCCATAGCTT			
C/EBPA	Forward: CCAAGAAGTCGGTGGACAAGAA	62	32	145
	Reverse: TCATTGTCACTGGTCAGCTCCA			

## Data Availability

The data used to support the findings of this study are available from the corresponding author upon request.

## References

[1] Hattemer-Frey H. A., Travis C. C. (1991). Benzo-a-pyrene: environmental partitioning and human exposure. *Toxicology and Industrial Health*.

[2] Machala M., Vondráček J., Bláha L., Ciganek M., Neča J.´. (2001). Aryl hydrocarbon receptor-mediated activity of mutagenic polycyclic aromatic hydrocarbons determined using in vitro reporter gene assay. *Mutation Research/Genetic Toxicology and Environmental Mutagenesis*.

[3] Dietrich C., Kaina B. (2010). The aryl hydrocarbon receptor (AhR) in the regulation of cell–cell contact and tumor growth. *Carcinogenesis*.

[4] Procházková J., Kabátková M., Bryja V. (2011). The interplay of the aryl hydrocarbon receptor and *β*-catenin alters both AhR-dependent transcription and Wnt/*β*-catenin signaling in liver progenitors. *Toxicological Sciences*.

[5] Albanes D., Jones D. Y., Micozzi M. S., Mattson M. E. (1987). Associations between smoking and body weight in the US population: analysis of NHANES II. *American Journal of Public Health*.

[6] Speciale A., Zena R., Calabrò C. (2018). Experimental exposure of blue mussels (*Mytilus galloprovincialis*) to high levels of benzo[*a*]pyrene and possible implications for human health. *Ecotoxicology and Environmental Safety*.

[7] Lin S., Fonteno S., Weng J. H., Talbot P. (2010). Comparison of the toxicity of smoke from conventional and harm reduction cigarettes using human embryonic stem cells. *Toxicological Sciences*.

[8] Rathore K., Cekanova M. (2015). Effects of environmental carcinogen benzo(a)pyrene on canine adipose-derived mesenchymal stem cells. *Research in Veterinary Science*.

[9] Zhou Y., Jiang R., An L. (2017). Benzo[*a*]pyrene impedes self-renewal and differentiation of mesenchymal stem cells and influences fracture healing. *Science of The Total Environment*.

[10] Dutta K., Ghosh D., Nazmi A., Kumawat K. L., Basu A. (2010). A common carcinogen benzo[a]pyrene causes neuronal death in mouse via microglial activation. *PLoS One*.

[11] Shimada T., Hiramatsu N., Kasai A. (2008). Suppression of adipocyte differentiation by *Cordyceps militaris* through activation of the aryl hydrocarbon receptor. *American Journal of Physiology-Endocrinology and Metabolism*.

[12] Whitlock J. P. (1999). Induction of cytochrome P4501A1. *Annual Review of Pharmacology and Toxicology*.

[13] Matsunawa M., Amano Y., Endo K. (2009). The aryl hydrocarbon receptor activator benzo[*a*]pyrene enhances vitamin D3 catabolism in macrophages. *Toxicological Sciences*.

[14] Davila J. C., Cezar G. G., Thiede M., Strom S., Miki T., Trosko J. (2004). Use and application of stem cells in toxicology. *Toxicological Sciences*.

[15] Kang K. S., Trosko J. E. (2011). Stem cells in toxicology: fundamental biology and practical considerations. *Toxicological Sciences*.

[16] Li B., Shao Q., Ji D., Li F., Chen G. (2015). Mesenchymal stem cells mitigate cirrhosis through BMP7. *Cellular Physiology and Biochemistry*.

[17] Bier A., Berenstein P., Kronfeld N. (2018). Placenta-derived mesenchymal stromal cells and their exosomes exert therapeutic effects in Duchenne muscular dystrophy. *Biomaterials*.

[18] Yu H., Du Y., Zhang X. (2014). The aryl hydrocarbon receptor suppresses osteoblast proliferation and differentiation through the activation of the ERK signaling pathway. *Toxicology and Applied Pharmacology*.

[19] Podechard N., Fardel O., Corolleur M., Bernard M., Lecureur V. (2009). Inhibition of human mesenchymal stem cell-derived adipogenesis by the environmental contaminant benzo(a)pyrene. *Toxicology In Vitro*.

[20] Choi Y. S., Park Y. B., Ha C. W. (2017). Different characteristics of mesenchymal stem cells isolated from different layers of full term placenta. *PLoS One*.

[21] Lee H. J., Jung J., Cho K. J., Lee C. K., Hwang S. G., Kim G. J. (2012). Comparison of in vitro hepatogenic differentiation potential between various placenta-derived stem cells and other adult stem cells as an alternative source of functional hepatocytes. *Differentiation*.

[22] Lager S., Powell T. L. (2012). Regulation of nutrient transport across the placenta. *Journal of Pregnancy*.

[23] Boitano A. E., Wang J., Romeo R. (2010). Aryl hydrocarbon receptor antagonists promote the expansion of human hematopoietic stem cells. *Science*.

[24] Lee J. M., Jung J., Lee H. J. (2012). Comparison of immunomodulatory effects of placenta mesenchymal stem cells with bone marrow and adipose mesenchymal stem cells. *International Immunopharmacology*.

[25] Page P., DeJong J., Bandstra A., Boomsma R. A. (2014). Effect of serum and oxygen concentration on gene expression and secretion of paracrine factors by mesenchymal stem cells. *International Journal of Cell Biology*.

[26] Barrientos S., Stojadinovic O., Golinko M. S., Brem H., Tomic-Canic M. (2008). Growth factors and cytokines in wound healing. *Wound Repair and Regeneration*.

[27] Lee J. Y., Kim E., Choi S. M. (2016). Microvesicles from brain-extract-treated mesenchymal stem cells improve neurological functions in a rat model of ischemic stroke. *Scientific Reports*.

[28] de Kok T. M. C. M., Driece H. A. L., Hogervorst J. G. F., Briedé J. J. (2006). Toxicological assessment of ambient and traffic-related particulate matter: a review of recent studies. *Mutation Research/Reviews in Mutation Research*.

[29] Sevastyanova O., Binkova B., Topinka J. (2007). In vitro genotoxicity of PAH mixtures and organic extract from urban air particles: part II: human cell lines. *Mutation Research/Fundamental and Molecular Mechanisms of Mutagenesis*.

[30] Hockley S. L., Arlt V. M., Brewer D. (2007). AHR- and DNA-damage-mediated gene expression responses induced by benzo(*a*)pyrene in human cell lines. *Chemical Research in Toxicology*.

[31] Andrysík Z., Vondráček J., Machala M. (2007). The aryl hydrocarbon receptor-dependent deregulation of cell cycle control induced by polycyclic aromatic hydrocarbons in rat liver epithelial cells. *Mutation Research/Fundamental and Molecular Mechanisms of Mutagenesis*.

[32] Nebert D. W., Roe A. L., Dieter M. Z., Solis W. A., Yang Y., Dalton T. P. (2000). Role of the aromatic hydrocarbon receptor and [*Ah*] gene battery in the oxidative stress response, cell cycle control, and apoptosis. *Biochemical Pharmacology*.

[33] Laberge R. M., Adler D., DeMaria M. (2013). Mitochondrial DNA damage induces apoptosis in senescent cells. *Cell Death & Disease*.

[34] Tomokiyo A., Maeda H., Fujii S. (2012). Alternation of extracellular matrix remodeling and apoptosis by activation of the aryl hydrocarbon receptor pathway in human periodontal ligament cells. *Journal of Cellular Biochemistry*.

[35] Herlin M., Öberg M., Ringblom J. (2015). Inhibitory effects on osteoblast differentiation in vitro by the polychlorinated biphenyl mixture Aroclor 1254 are mainly associated with the dioxin-like constituents. *Toxicology In Vitro*.

[36] Ryan E. P., Holz J. D., Mulcahey M., Sheu T. J., Gasiewicz T. A., Puzas J. E. (2007). Environmental toxicants may modulate osteoblast differentiation by a mechanism involving the aryl hydrocarbon receptor. *Journal of Bone and Mineral Research*.

[37] Heo J. S., Lee S. G., Kim H. O. (2017). Distal-less homeobox 5 is a master regulator of the osteogenesis of human mesenchymal stem cells. *International Journal of Molecular Medicine*.

